# Surgical treatment of peroneal calcific tendinitis in an uncommon localization: A case report

**DOI:** 10.1002/ccr3.2709

**Published:** 2020-02-11

**Authors:** Anas Taha, Saulius Adamonis, Stephanie Taha‐Mehlitz, Andreas Maurer

**Affiliations:** ^1^ Surgery Department Kantonsspital Winterthur Winterthur Switzerland; ^2^ Clinic for General and Visceral Surgery Spital Einsiedeln Einsiedeln Switzerland; ^3^ Clarunis Universitäres Bauchzentrum University Hospital Basel Basel Switzerland; ^4^ Clinic for Traumatology, Orthopedics and Hand Surgery Spital Laufenburg Laufenburg Switzerland

**Keywords:** calcific tendinitis, calcifications, peroneus longus tendon, tendinitis

## Abstract

Calcific tendinitis is a common pathology in the shoulder but is uncommon in the peroneus longus tendon, which is considered when patient presents with a palpable tenderness without signs of inflammation. Differential diagnosis is important, as the condition is often overlooked. Conservative treatment has proven successful, though surgery might be an alternative.

## Graphical abstract

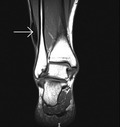

## 1 INTRODUCTION

Calcific tendinitis is an uncommon pathology of a tendon. The current study reports a case of a 58‐year‐old woman with an uncomfortable swelling and local tenderness in the lateral part of the right lower extremity. After diagnostic MRI, a surgical excision was performed. Pain resolved after surgery.

Calcific tendinitis is a disorder characterized by deposits of hydroxyapatite (a crystalline calcium phosphate) in any tendon of the body. A common localization of calcific tendinitis is in the tendons of the rotator cuff, especially in the supraspinatus and infraspinatus tendons. In the lower extremity, calcifications are rare and are usually found in the patellar and Achilles tendons or in the greater trochanter.^1^ The calcific deposits may be located within the tendon or in the soft tissues adjacent to the tendon or ligament near its attachment to the bone.^2^ The deposits most often occur in females aged 40‐60 years.^3^

A comprehensive literature review was performed by searching the PubMed‐National Center for Biotechnology Information database using the keywords ‘calcific, tendinitis AND peroneus’. The search yielded 5 publications, 1 of which was on ossification, while another was on hip pain. One case report of calcific tendinitis was found in the Elsevier bibliotheca. In total, 4 case reports on calcific tendinitis of the peroneus tendon were found and included in our literature review (Table 1).

**Table 1:** Literature review of calcific tendinitis of the peroneus tendon

Reference	Gender	Age, y	Examination	Treatment
1	F	50	Radiograph	Steroid injection (Depomedrone 40 mg mixed with local anesthesia)
4	M	32	Radiograph + Blood test	Steroid injection (3 mg Betamethasone Sodium Phosphate + 3 mg Betamethasone Dipropionate mixed with 3 ml xylocaine)
5	F	22	Radiograph + MRI	NSAIDs, rest and supportive footwear
6	M	46	Radiograph + Blood test	NSAIDs, strict bed rest and limb elevation
Our case report	F	58	MRI	Surgical excision

Note

Reference in the text: “4 case reports on calcific tendinitis of the peroneus tendon were found and included in our literature review (Table 1).”

Abbreviations: F, female; M, male; NSAIDs, nonsteroidal anti‐inflammatory drugs.

**Figure 1:**
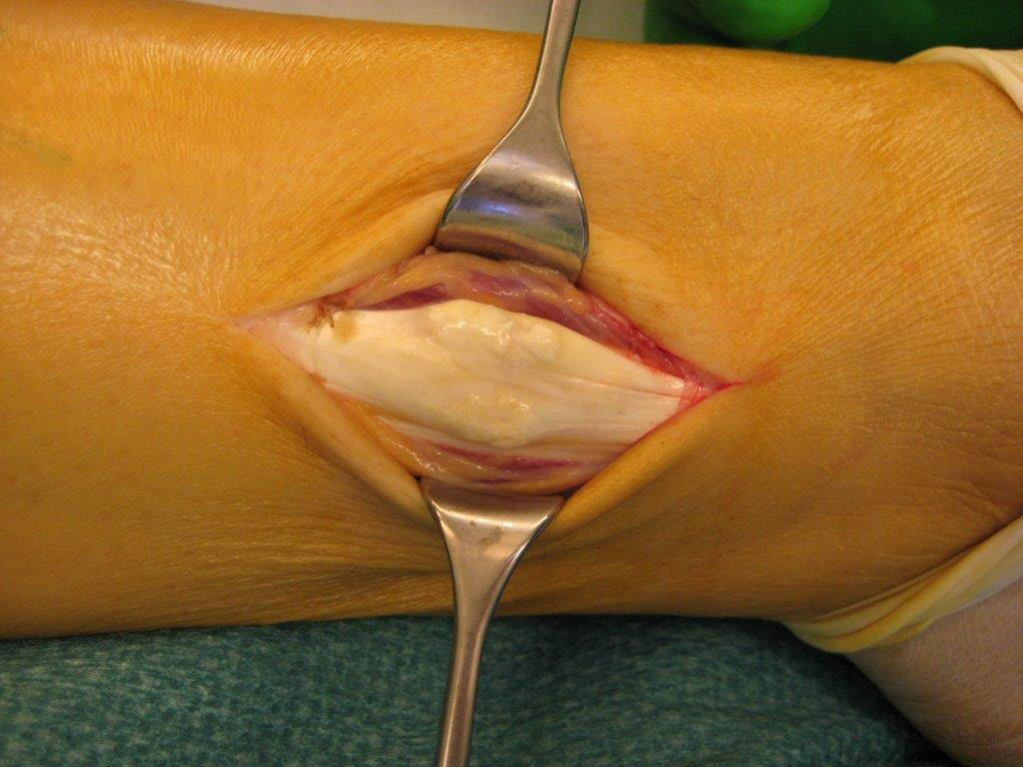
Intraoperative view of the calcification of the peroneal tendon in the musculotendinous transition in the right lower extremity

To the best of our knowledge, only four case reports of calcific tendinitis have been published in the English literature. We present a case of a 58‐year‐old woman with peroneal calcific tendinitis in the musculotendinous transition (Figure 1).

## 2 CASE PRESENTATION

**Figure 2:**
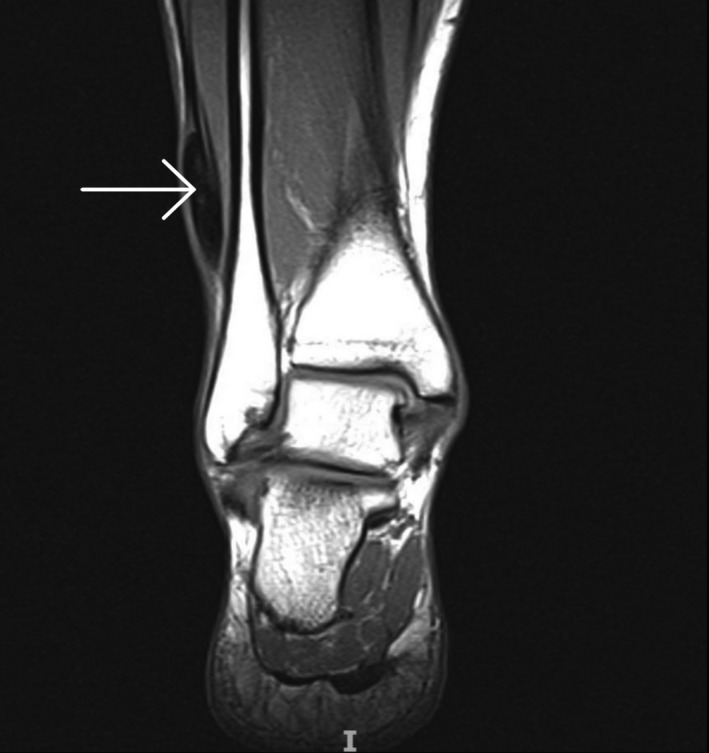
T1 sequence on the MRI showing a hypointense area of 3 × 1 cm in the medial lower extremity

**Figure 3:**
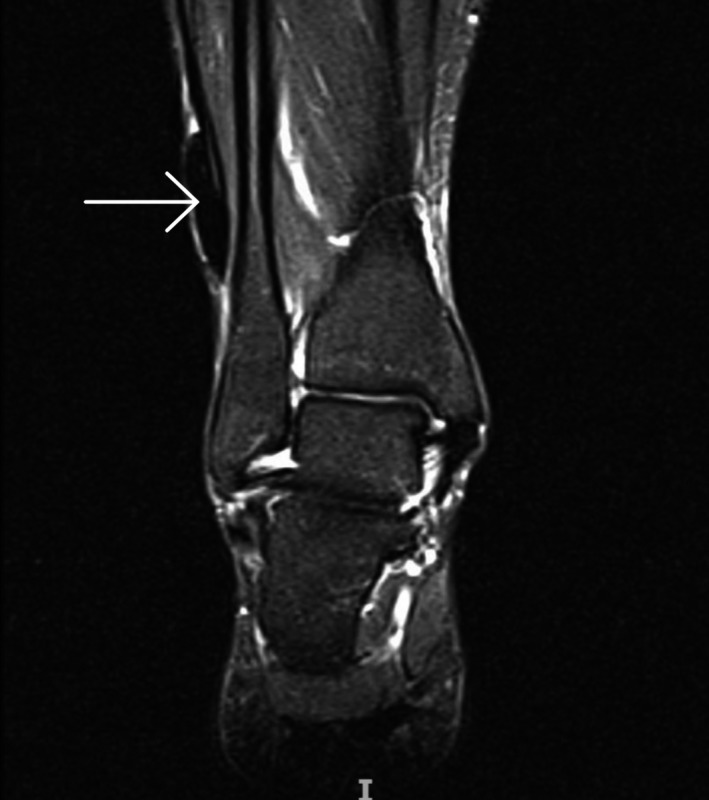
T2 sequence on the MRI showing a hypointense area of 3 × 1 cm in the medial lower extremity

**Figure 4:**
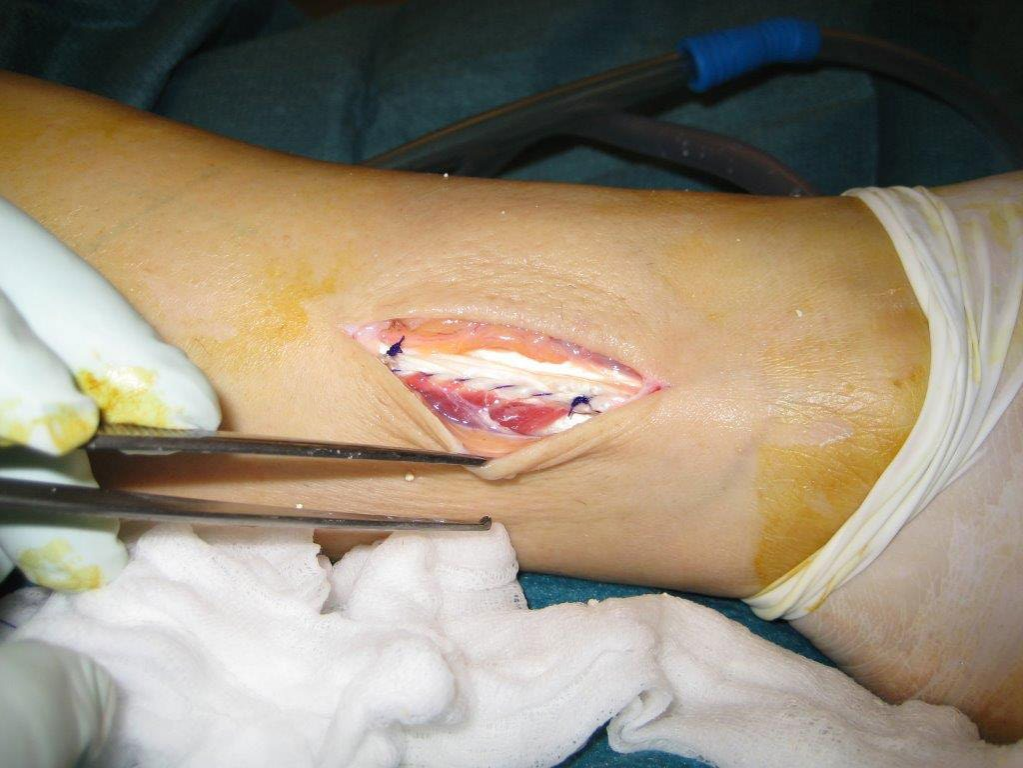
Intraoperative result after excision of the calcifications and tendon reconstruction

A 58‐year‐old woman who had been riding a horse for more than 30 years presented with swelling with local tenderness in the lateral part of the right lower extremity. The swelling had increased over the past 10 years but only recently affected her daily life. The swelling had no signs of infection. The patient was not able to continue sports due to an unpleasant sensation in the ankle when moving. In her past medical history, the patient had a mild right ankle joint distortion 40 years ago. In the clinical examination, the mobility of the ankle joint was not restricted. Beneath the swelling, there was a hard, displaceable nodulus approximately 6 cm proximal to the lateral malleolus. We decided to perform an MRI instead of X‐ray so we could differentiate between intramuscular tumors due to the uncommon localization of the symptoms in the proximal end of the peroneus tendon. The MRI revealed a hypointense area of 3x1 cm in T1 and T2 as signs of calcification (Figures 2, 3). Because of the athletic nature of the patient and her desire for a faster recovery, we performed surgical treatment through a 4‐cm‐long skin incision above the perineal tendon. The calcification was closely adherent to the tendon, and approximately 50% of the tendon was destroyed. We removed the calcification and reconstructed the remaining peroneal tendon (Figure 4). Histological examination of the resected tendon confirmed the calcification. The postoperative course was uneventful, and her pain resolved after surgery. Postoperatively, the patient was able to continue sports with no restrictions after 1 week of physical therapy. In the follow‐up 1 and 5 years after surgery, the patient had no discomfort and was able to ride a horse again.

## 3 DISCUSSION AND CONCLUSIONS

The etiology of tendinous calcifications remains unknown. Tendinous calcifications may be a consequence of micro‐tears or intra‐tendinous ischemia. Healing of tendon injuries due to calcification, direct local stress necrosis or through fatty acid and soap intermediaries and local hypoxia secondary to either mechanical or vascular factors has been proposed to proceed through causative factors.^7^

Uhthoff and Loehr^8^ described a three‐phase process: precalcific, calcific (divided into three subphases: formative, resting, and resorptive), and postcalcific. In the precalcific phase, tenocytes transform into chondrocytes; the calcification phase is characterized by the formation of calcium deposits; the stable phase is characterized by the presence of calcifications and symptoms; and the resorptive phase includes a period of spontaneous resorption during which there is both neoangiogenesis, beginning at the margin of the deposit, and phagocyte infiltration.

Calcific tendinitis can usually be diagnosed on a simple radiograph. Sharply outlined or fluffy and heterogeneous deposits may be seen. Another diagnostic choice is ultrasonography, which is as sensitive as X‐ray.^9^ MRI is a good imaging method for making a better differential diagnosis of an uncommon localization of the calcific tendinitis in the musculotendinous transition. In T1 and T2 sequences, hypointense signal of calcific deposits is seen.

A differential diagnosis between calcification and ossification is important for planning conservative or surgical treatments. On the radiography, calcifications normally appear as mineralized densities, whereas mature bone has an outer cortex and an inner trabecular pattern. CT is more sensitive in detecting calcifications and ossification.^10^ In sonography, it is difficult to differentiate between ossifications and calcifications, especially when acoustic shadowing is present. Calcifications usually have a uniformly low signal intensity on all MRI sequences. Mature bone shows bone marrow within trabecular spaces on both CT and MRI. However, immature bone is not well organized and may be more difficult to discriminate from calcifications.^10^

The initial conservative treatments of calcific tendinitis include rest, physical therapy, nonsteroidal anti‐inflammatory drugs, and extracorporeal shock wave therapy (ESWT). ESWT may be considered if no improvement is observed with other nonoperative techniques. It appears that the effect of calcium disintegration or alterations in the deposit's consistency is derived from direct energy transfer. Observed trends show a better dissolution rate with high‐energy waves for single solid deposits.^11^

Surgical treatment is considered after 6 months if symptoms progress or fail to improve with conservative management or earlier if activities of daily living are significantly impacted. In cases of calcific tendinitis of the shoulder, arthroscopic surgery is preferred.

Our case is important because in our case report, calcific tendinitis was found in the proximal part of the peroneus longus tendon, whereas the four other case reports described the involvement of the distal part of the tendon. A possible etiology could have been micro‐tears due to horse riding because of permanent dorsal flexion of the foot in the stapes.

In our case, due to the calcific depositions in the peroneus tendon, open surgery was performed. Surgery was chosen as the management of choice in our case, as the symptoms impaired the daily life of the patient. Our case report is the only one with surgery as a treatment, but this option was more acceptable for the patient.

Calcifications of the peroneus longus tendon are very rare, but this diagnosis should be considered when a patient presents with a hard‐palpable swelling. Surgery is a good option for treating calcific tendinitis with no risk of recurrence.

## CONFLICT OF INTEREST

None declared.

## AUTHOR CONTRIBUTIONS

AT and STM: conceived of this case report. AT and SA: wrote the first draft of the manuscript. AM: revised the final manuscript. All the authors have read and approved the manuscript.

## INFORMED CONSENT

Informed patient consent was obtained.
